# Gene array and real time PCR analysis of the adrenal sensitivity to adrenocorticotropic hormone in pig

**DOI:** 10.1186/1471-2164-9-101

**Published:** 2008-02-27

**Authors:** Dominique Hazard, Laurence Liaubet, Magali SanCristobal, Pierre Mormède

**Affiliations:** 1 Laboratoire PsyNuGen, INRA UMR1286, CNRS UMR5226, Université de Bordeaux 2, 146 rue Léo-Saignat, F-33076 Bordeaux, France; 2 Laboratoire de Génétique Cellulaire, INRA UMR444, F-31326 Castanet-Tolosan, France

## Abstract

**Background:**

Variability in hypothalamic-pituitary-adrenal (HPA) axis activity has been shown to be influenced by genetic factors and related to great metabolic differences such as obesity. The aim of this study was to investigate molecular bases of genetic variability of the adrenal sensitivity to ACTH, a major source of variability, in Meishan (MS) and Large White (LW) pigs, MS being reported to exhibit higher basal cortisol levels, response to ACTH and fatness than LW. A pig cDNA microarray was used to identify changes in gene expression in basal conditions and in response to ACTH stimulation.

**Results:**

Genotype and/or ACTH affected the expression of 211 genes related to transcription, cell growth/maintenance, signal transduction, cell structure/adhesion/extra cellular matrix and protein kinase/phosphatase activity. No change in the expression of known key regulator proteins of the ACTH signaling pathway or of steroidogenic enzymes was found. However, *Mdh2*, *Sdha*, *Suclg2*, genes involved in the tricarboxylic acid (TCA) pathway, were over-expressed in MS pigs. Higher TCA cycle activity in MS than in LW may thus result in higher steroidogenic activity and thus explain the typically higher cortisol levels in MS compared to LW. Moreover, up-regulation of *Star *and *Ldlr *genes in MS and/or in response to ACTH suggest that differences in the adrenal function between MS and LW may also involve mechanisms requisite for cholesterol supply to steroidogenesis.

**Conclusion:**

The present study provides new potential candidate genes to explain genetic variations in the adrenal sensitivity to ACTH and better understand relationship between HPA axis activity and obesity.

## Background

The hypothalamic-pituitary-adrenal (HPA) axis and more particularly the adrenal gland constitute a principal node of the mammalian endocrine system. The main function of the adrenal cortex is to produce glucocorticoids (cortisol in pig) and mineralocorticoids under the influence of pituitary adrenocorticotropic hormone (ACTH). Adrenal hormones, essential for survival, play important roles in stress responses, metabolism regulation, immunity, reproduction, water and salt balance and various brain functions [1].

Large variations in HPA axis activity (i.e. basal and in response to stress) have been related to genetic factors (in human [2], in mice [3], in rats [4-6], in pigs [7] and in birds [8-10]). Variations in HPA axis activity are also related to important metabolic differences. For example, human abdominal obesity has been associated with alterations in HPA axis functioning [11,12]. In a recent experiment comparing five genetic stocks of pigs (Large White, Landrace, Duroc, Meishan and Piétrain), a positive relationship between cortisol levels in urine (basal and after transportation stress) and body fat content was found both within and across breeds [13]. In addition, Meishan pigs have been reported to exhibit higher basal cortisol levels, response to ACTH and body fat content than Large White pigs [7,14]. Meishan and Large White lines of pigs thus constitute a valuable biological model to investigate the relationship between HPA axis activity and metabolic regulation.

Among the different genetic mechanisms involved in HPA axis activity variability [15], several experimental findings suggest that sensitivity to ACTH is a major target in human [16] and in rats [4,17]. In the pig, genetic-based differences in cortisol secretion were shown in response to corticotropin-releasing hormone (CRH) although the ACTH response did not differ among individuals [18]. Moreover, metabolic clearance of cortisol bears no relationship to the cortisol response to ACTH [19]. Previous findings [14] indicate that the difference in HPA axis activity between LW and MS pigs may originate from the adrenal gland although these breeds also differ in corticosteroid-binding globulin (CBG) levels that influence circulating levels of cortisol [20]. In this study, we explore the molecular mechanism responsible for the difference in adrenal sensitivity to ACTH in MS and LW pigs.

The actions of ACTH in the adrenal cortex are mediated via two temporally distinct pathways. Acute and chronic regulation of steroidogenesis occur within minutes and hours, respectively [21,22]. The acute response is initiated by the mobilization and delivery of the substrate, cholesterol, for steroid hormone biosynthesis from the outer to the inner mitochondrial membrane, where it is metabolized to pregnenolone by the cytochrome P450 cholesterol side chain cleavage enzyme (P450scc) [23]. The slower, long-lasting response to ACTH directs transcription of genes encoding the steroidogenic enzymes [22,24]. Most studies of ACTH action have focused on the long term induction (several hours) of transcripts either by investigating transcripts with specific functions in steroidogenesis [22,24] or by genome-wide analysis [25-27]. However, the acute response to ACTH stimulation has been reported to require *de novo *protein synthesis (for review see ref. [21]). The steroidogenic acute regulatory protein (StAR), that is responsible for the transfer of cholesterol from the outer to the inner mitochondrial membrane, has been proposed to be the rate-limiting and regulated step in steroidogenesis [23,28]. Changes in *Star *gene expression induced by ACTH have been observed as early as 30 min and were maximally elevated between 1 and 3 h [29]. On the other hand, cortisol responses to ACTH injections in Meishan and Large White pigs have been reported to be at maximum levels at 1 hour [14,30]. Taking together these findings, we hypothesized that transcriptional regulation may in fact take place in the acute response to ACTH, i.e. within 1 hour, in both lines of pigs. Differences in transcriptional regulation at the adrenal gland could already exist between both genotypes in basal conditions because basal cortisol levels are typically greater in MS pigs.

Considering the findings described above and the literature concerning acute regulation of transcripts by ACTH in adrenals, we undertook a microarray analysis of gene expression in the adrenal glands of MS and LW pigs under basal conditions and in response to acute stimulation by ACTH. The aim of this study was to investigate the molecular bases of the genetic differences in adrenal sensitivity to ACTH. We found that genotype and/or ACTH affected the expression of 211 genes which provide new potential candidate genes to explain genetic variations in the adrenal sensitivity to ACTH.

## Results

### Cortisol levels

**Figure 1:**
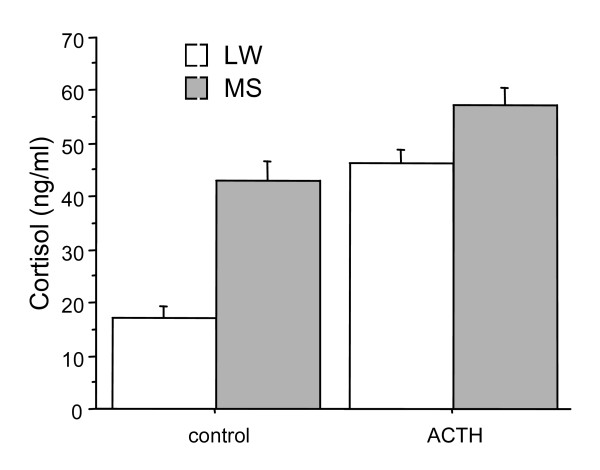
**Cortisol concentrations (ng/ml plasma)**. Cortisol concentrations (ng/ml plasma) measured in Large White (LW) and Meishan (MS) piglets either untreated (control) or 1 hour after injection (ACTH) of a high dose (250 μg per animal) of 1–24 ACTH (Immediate synacthen). (n = 6 per experimental group, means ± SE).

Comparison of plasma cortisol concentrations (figure 1) in control and ACTH-injected pigs showed significant breed (p < 0.0001) and treatment (p < 0.0001) effects and significant interaction (p ≤ 0.001). In control animals, basal cortisol levels were higher in Meishan pigs than in Large White pigs. Injection of ACTH increased cortisol levels in both genotypes but to a larger extent in Large White than in Meishan pigs.

### Gene transcript regulation by genotype and ACTH treatment

Using the normalization process described in the methods section, 3496 of the 8959 transcripts present on the pig cDNA array (approximately 40%) were found to be expressed in adrenal glands in our experimental conditions. Using the criterion of < 5% FDR, 241 transcripts were identified to be significantly up-regulated or downregulated by genotype and/or ACTH challenge (see additional file 1). Among these, 211 transcripts corresponded to unique annotated transcripts (five present twice) and 25 remained unknown. These 241 transcripts were categorized according to the genotype and/or treatment effect and ordered by absolute fold change. Table 1 lists transcripts most significantly affected by genotype and/or treatment at p < 0.0001. Among these, 51 transcripts were significantly affected by genotype (39 transcripts up-regulated and 12 down regulated in Meishan), 21 transcripts were significantly affected by ACTH treatment (12 transcripts up-regulated and six down regulated in response to ACTH treatment) and 36 transcripts were significantly affected by both genotype and ACTH treatment. The major functional categories for these genes included transcription regulation, cell growth/maintenance, signal transduction, structural/cell adhesion/ECM and protein kinase activity.

**Table 1:** Genes differentially expressed depending on genotype and/or ACTH treatment

Gene symbol	Gene name	Gene Function	p-value (ANOVA)			Fold Change			
						
						genotype: MS/LW		treatment: ACTH/control	
			
			genotype	treatment	interaction	control	ACTH	LW	MS
**Genes up-regulated in Meishan pigs**									

Rnf2	ring finger protein 2	Transcription regulation	2.81E-13	7.89E-01	1.96E-02	3.5	2.5	1.2	-1.2
Mdk	midkine (neurite growth-promoting factor 2)	Cell growth/maintenance	4.97E-06	NS	NS	2.0	1.5	1.2	-1.1
Snf1lk	SNF1-like kinase	Protein Kinase activity	8.19E-05	NS	NS	1.8	1.8	1.2	1.3
Ccnd1	cyclin D1	Cell growth/maintenance	9.83E-06	NS	NS	1.7	1.5	1.1	1.0
Cd99	CD99 molecule	Structural/Cell adhesion/ECM	1.01E-05	NS	NS	1.7	1.6	1.0	1.0
Col21a1	collagen, type XXI, alpha 1	Structural/Cell adhesion/ECM	9.39E-05	NS	NS	1.7	1.5	-1.0	-1.2
Gprc5b	G protein-coupled receptor, family C, group 5, member B	Signal transduction (glutamatergic)	9.53E-07	NS	NS	1.6	1.5	1.1	-1.0
Ldhd	lactate dehydrogenase D	ATP biosynthesis	3.77E-06	NS	NS	1.6	1.6	1.1	1.1
Ppp1r1a	protein phosphatase 1, regulatory (inhibitor) subunit 1A	Phosphatase inhibitor	8.18E-06	NS	NS	1.6	1.4	-1.0	-1.2
Mrps6	mitochondrial ribosomal protein S6	Translation	1.25E-06	NS	NS	1.6	1.4	1.1	-1.1
Traf5	TNF receptor-associated factor 5	Signal transduction	5.80E-05	NS	NS	1.5	1.5	1.0	1.0
Cse1l	CSE1 chromosome segregation 1-like (yeast)	Protein transport	2.98E-06	NS	NS	1.5	1.4	1.1	1.0
Hist2h2aa	histone cluster 2, H2aa4	Nucleosome Assembly	2.10E-05	NS	NS	1.5	1.5	-1.1	-1.1
Cd99	CD99 molecule	Structural/Cell adhesion/ECM	2.27E-05	NS	NS	1.5	1.6	-1.1	1.0
Mif	macrophage migration inhibitory factor (glycosylation-inhibiting factor)	Cell growth/maintenance	1.25E-05	NS	NS	1.5	1.3	1.1	-1.0
Ptpmt1	protein tyrosine phosphatase, mitochondrial 1	Protein Phosphatase activity	1.73E-08	NS	NS	1.5	1.5	1.0	1.1
Mdh2	malate dehydrogenase 2, NAD (mitochondrial)	Tricarboxylic acid cycle	2.59E-06	NS	NS	1.5	1.5	1.0	1.1
Malat1	metastasis associated lung adenocarcinoma transcript 1 (non coding RNA)	unknown	4.07E-05	NS	NS	1.5	1.3	-1.0	-1.2
Suclg2	succinate-CoA ligase, GDP-forming, beta subunit	Tricarboxylic acid cycle	1.82E-05	NS	NS	1.4	1.3	1.0	-1.1
Eif3s7	eukaryotic translation initiation factor 3, subunit 7 zeta, 66/67kDa	Translation regulation	4.61E-05	5.87E-01	2.64E-02	1.4	1.1	1.1	-1.2
Magi3	membrane associated guanylate kinase, WW and PDZ domain containing 3	Protein Kinase activity	2.40E-05	NS	NS	1.4	1.8	-1.2	1.0
Ppap2b	phosphatidic acid phosphatase type 2B	Protein Phosphatase activity	3.45E-05	6.74E-02	4.48E-02	1.4	2.2	-1.0	1.5
Cys1	cystin 1	unknown	1.08E-08	NS	NS	1.4	1.5	-1.1	-1.0
Txnl2	thioredoxin-like 2	Electron transport	5.63E-05	NS	NS	1.4	1.3	1.0	1.0
C8orf53	chromosome 8 open reading frame 53	unknown	1.34E-05	NS	NS	1.4	1.4	1.1	1.1
C14orf2	chromosome 14 open reading frame 2	unknown	1.65E-05	NS	NS	1.4	1.3	1.0	-1.0
Dusp26	dual specificity phosphatase 26 (putative)	unknown	3.78E-06	NS	NS	1.4	1.4	1.0	1.0
Sod2	superoxide dismutase 2, mitochondrial	Super oxide metabolism	1.61E-06	NS	NS	1.4	1.5	-1.1	-1.0
Ssr3	signal sequence receptor, gamma (translocon-associated protein gamma)	Cotranslational membrane targeting	7.82E-05	NS	NS	1.4	1.3	1.1	1.0
Xpnpep1	X-prolyl aminopeptidase (aminopeptidase P) 1, soluble	Proteolysis	3.05E-05	NS	NS	1.3	1.3	-1.0	-1.0
Hint1	histidine triad nucleotide binding protein 1	Signal transduction	6.24E-05	NS	NS	1.3	1.2	1.1	1.0
Hsbp1	heat shock factor binding protein 1	Transcription regulation	6.13E-05	NS	NS	1.3	1.2	1.0	-1.0
Mrps10	mitochondrial ribosomal protein S10	Structural constituent of ribosome	6.05E-05	NS	NS	1.3	1.3	-1.0	1.0
Lpp	LIM domain containing preferred translocation partner in lipoma	unknown	4.71E-05	NS	NS	1.2	1.3	1.1	1.1
Pgk1	phosphoglycerate kinase 1	Protein Kinase activity	1.83E-05	NS	NS	1.2	1.4	-1.1	1.1
Ppap2d	phosphatidic acid phosphatase type 2d	Protein Phosphatase activity	6.15E-06	NS	NS	1.2	1.3	-1.0	1.1
Atp5b	ATP synthase, H+ transporting, mitochondrial F1 complex, beta polypeptide	ATP biosynthesis	1.07E-05	NS	NS	1.2	1.1	1.0	-1.0
Ccny	cyclin Y	unknown	1.59E-05	NS	NS	1.2	1.3	1.0	1.1
Cox6b1	cytochrome c oxidase subunit Vib polypeptide 1 (ubiquitous)	Oxydoreductase activity	9.02E-05	NS	NS	1.2	1.3	-1.0	1.1

**Genes down-regulated in Meishan pigs**									

C1qa	complement component 1, q subcomponent, A chain	Cell-cell signaling	3.08E-06	NS	NS	-2.1	-1.5	-1.1	1.2
Cited1	Cbp/p300-interacting transactivator, with Glu/Asp-rich carboxy-terminal domain, 1	Transcription regulation	1.88E-10	NS	NS	-1.8	-2.0	1.1	-1.0
Tmem14c	transmembrane protein 14C	Structural/Cell adhesion/ECM (mito)	4.24E-06	4.81E-01	4.54E-02	-1.8	-1.3	-1.2	1.1
Hmgb3	high-mobility group box 3	Transcription regulation	2.00E-08	NS	NS	-1.6	-1.4	-1.2	-1.0
Tmem14c	transmembrane protein 14C	Structural/Cell adhesion/ECM (mito)	5.40E-09	3.37E-01	1.61E-02	-1.6	-1.3	-1.1	1.1
Bphl	biphenyl hydrolase-like (serine hydrolase; breast epithelial mucin-associated antigen)	Amino acid metabolism	2.71E-06	NS	NS	-1.5	-1.4	-1.1	-1.0
Ttll12	tubulin tyrosine ligase-like family, member 12	unknown	8.52E-08	NS	NS	-1.5	-1.6	1.1	-1.0
Gpx3	glutathione peroxidase 3 (plasma)	Peroxidase activity	8.06E-08	NS	NS	-1.5	-1.5	-1.0	-1.1
Fkbp4	FK506 binding protein 4, 59kDa	Protein binding	4.64E-05	NS	NS	-1.4	-1.3	1.0	1.1
Gns	glucosamine (N-acetyl)-6-sulfatase (Sanfilippo disease IIID)	Glycosaminoglycan catabolism	5.48E-05	NS	NS	-1.3	-1.5	1.1	-1.1
Nr1h2	nuclear receptor subfamily 1, group H, member 2	Transcription regulation	1.30E-05	NS	NS	-1.2	-1.3	1.0	-1.1
H2afj	H2A histone family, member J	Nucleosome Assembly	3.50E-05	NS	NS	-1.2	-1.3	-1.0	-1.1

**Genes up-regulated by ACTH**									

Bag3	BCL2-associated athanogene 3	Cell growth/maintenance	3.76E-01	9.34E-11	5.19E-03	1.2	-1.3	3.0	2.0
Ier3	immediate early response 3	Cell growth/maintenance	4.49E-01	2.83E-09	3.81E-02	1.3	-1.1	2.6	1.9
Adamts1	ADAM metallopeptidase with thrombospondin type 1 motif, 1	Cell growth/maintenance	NS	2.37E-06	NS	1.4	-1.1	2.6	1.7
Gadd45a	growth arrest and DNA-damage-inducible, alpha	Regulation of protein kinase activity	NS	1.46E-07	NS	1.1	-1.1	2.1	1.7
Id3	inhibitor of DNA binding 3, dominant negative helix-loop-helix protein	Transcription regulation	NS	4.69E-07	NS	-1.1	-1.2	2.0	1.9
Tfpi2	tissue factor pathway inhibitor 2	Structural/Cell adhesion/ECM	NS	4.72E-07	NS	1.1	1.0	1.8	1.7
Odc1	ornithine decarboxylase 1	Cell growth/maintenance	NS	2.85E-06	NS	1.2	1.0	1.6	1.4
Tomm20	translocase of outer mitochondrial membrane 20 homolog (yeast)	Protein transport (mb ext mito)	NS	9.51E-05	NS	1.2	1.1	1.5	1.4
Timp1	TIMP metallopeptidase inhibitor 1	Cell growth/maintenance	NS	2.88E-07	NS	1.0	-1.1	1.4	1.3
Ddx3x	DEAD (Asp-Glu-Ala-Asp) box polypeptide 3, X-linked	RNA helicase activity	NS	1.19E-05	NS	1.1	1.0	1.4	1.3
C10orf46	chromosome 10 open reading frame 46	unknown	NS	6.70E-06	NS	1.0	1.2	1.4	1.6
Gnl2	guanine nucleotide binding protein-like 2 (nucleolar)	unknown	NS	3.73E-05	NS	1.1	-1.0	1.3	1.2

**Genes down regulated by ACTH**									

Stat5a	signal transducer and activator of transcription 5A	Transcription regulation	NS	8.12E-05	NS	-1.0	1.0	-1.4	-1.3
Wasf2	WAS protein family, member 2	Signal transduction (G-protein)	NS	7.81E-05	NS	-1.0	1.0	-1.3	-1.3
Unc84b	unc-84 homolog B (C. elegans)	Cell growth/maintenance	NS	4.26E-05	NS	1.2	-1.0	-1.3	-1.6
Cntf	ciliary neurotrophic factor	Signal transduction	NS	4.93E-05	NS	-1.0	1.1	-1.3	-1.2
Nfe2l2	nuclear factor (erythroid-derived 2)-like 2	Transcription regulation	NS	5.59E-06	NS	1.1	1.0	-1.3	-1.3
Pcbd1	pterin-4 alpha-carbinolamine dehydratase/dimerization cofactor of hepatocyte nuclear factor 1 alpha (TCF1)	Transcription regulation	NS	5.70E-05	NS	1.1	1.0	-1.2	-1.3
Bmpr2	bone morphogenetic protein receptor, type II (serine/threonine kinase)	Protein Kinase activity	NS	3.15E-05	NS	-1.1	1.0	-1.2	-1.1
Ctnnb1	catenin (cadherin-associated protein), beta 1, 88kDa	Transcription regulation	NS	1.01E-05	NS	-1.0	-1.1	-1.2	-1.2
Gdf8	growth differentiation factor 8	Signal transduction (TGFb)	NS	5.41E-05	NS	1.1	-1.1	-1.2	-1.3

**Genes upregulated in Meishan and by ACTH**									

Crem	cAMP responsive element modulator	Transcription regulation	4.34E-02	2.70E-10	NS	1.7	1.1	4.4	3.0
Cd83	CD83 molecule	Signal transduction	6.77E-06	3.80E-10	NS	1.9	1.5	3.2	2.6
Eif1b	eukaryotic translation initiation factor 1B	Translation regulation	9.08E-06	7.89E-10	NS	1.5	1.9	2.5	3.3
Tob1	transducer of ERBB2, 1	Transcription regulation	4.11E-02	6.96E-08	NS	1.1	1.0	2.5	2.3
Ldlr	low density lipoprotein receptor (familial hypercholesterolemia)	Cholesterol mobilization	5.19E-03	4.15E-07	NS	1.6	1.3	2.3	1.8
Chchd2	coiled-coil-helix-coiled-coil-helix domain containing 2	unknown	4.72E-04	3.36E-06	NS	1.4	1.3	1.6	1.5
Ckb	creatine kinase, brain	Structural/Cell adhesion/ECM	1.03E-06	3.00E-02	NS	2.1	1.9	1.3	1.2
Mki67ip	MKI67 (FHA domain) interacting nucleolar phosphoprotein	rRNA metabolism	1.25E-04	9.14E-05	NS	1.3	1.3	1.3	1.3
Maob	monoamine oxidase B	Electron transport	4.43E-05	4.23E-03	NS	1.4	1.3	1.2	1.2
Dnaja	DnaJ (Hsp40) homolog, subfamily A, member 2	Cell growth/maintenance	1.59E-05	2.55E-03	NS	1.5	1.2	1.2	1.0
H19	H19, imprinted maternally expressed untranslated mRNA	unknown	9.02E-07	5.79E-03	NS	1.5	1.6	1.1	1.3
Dleu1	deleted in lymphocytic leukemia, 1	Cell growth/maintenance	4.90E-07	2.14E-03	NS	1.5	2.0	1.1	1.4

**Genes up-regulated in Meishan and down-regulated by ACTH**									

Selenbp1	selenium binding protein 1	Transporter activity	2.98E-05	1.41E-02	NS	1.3	1.5	-1.3	-1.1
C16orf33	chromosome 16 open reading frame 33	unknown	5.03E-05	3.98E-02	NS	1.2	1.3	-1.2	-1.0
Ech1	enoyl Coenzyme A hydratase 1, peroxisomal	Fatty acid metabolism	5.27E-07	6.31E-03	NS	1.4	1.4	-1.2	-1.2
Anpep	alanyl (membrane) aminopeptidase (aminopeptidase N, aminopeptidase M, microsomal aminopeptidase, CD13, p150)	Development/Morphogenesis	5.65E-12	8.27E-03	NS	2.1	1.9	-1.1	-1.2
Iscu	iron-sulfur cluster scaffold homolog (E. coli)	unknown	6.09E-05	1.03E-03	NS	1.3	1.2	-1.1	-1.2
Enpp2	ectonucleotide pyrophosphatase/phosphodiesterase 2 (autotaxin)	Signal transduction (G-protein)	8.22E-05	6.17E-03	NS	1.4	1.3	-1.1	-1.2
Klhl22	kelch-like 22 (Drosophila)	unknown	2.75E-04	9.94E-05	NS	1.3	1.1	-1.1	-1.2
Sdha	succinate dehydrogenase complex, subunit A, flavoprotein (Fp)	Tricarboxylic acid cycle	3.38E-05	3.08E-02	NS	1.2	1.2	-1.0	-1.1

**Genes down regulated in Meishan and by ACTH**									

Ctdsp2	CTD (carboxy-terminal domain, RNA polymerase II, polypeptide A) small phosphatase 2	unknown	1.84E-02	4.68E-08	NS	-1.1	-1.3	-1.6	-1.8
Pecam1	platelet/endothelial cell adhesion molecule (CD31 antigen)	Signal transduction	2.74E-05	8.45E-04	NS	-1.6	-1.6	-1.4	-1.5
Dab2	disabled homolog 2, mitogen-responsive phosphoprotein (Drosophila)	Cell growth/maintenance	2.92E-02	5.63E-05	NS	-1.1	-1.3	-1.4	-1.7
Cyb5b	cytochrome b5 type B (outer mitochondrial membrane)	Transporter activity	4.15E-03	2.59E-06	NS	-1.1	-1.3	-1.4	-1.6
Ostf1	osteoclast stimulating factor 1	Signal transduction	1.66E-07	1.08E-02	NS	-1.7	-1.5	-1.3	-1.1
Gdf8	growth differentiation factor 8	Signal transduction (TGFb)	4.95E-03	6.71E-05	NS	-1.1	-1.3	-1.3	-1.4
Nfat5	nuclear factor of activated T-cells 5, tonicity-responsive	Transcription regulation	2.75E-03	1.27E-05	NS	-1.2	-1.2	-1.3	-1.3
Acox1	acyl-Coenzyme A oxidase 1, palmitoyl	Fatty acid metabolism	7.62E-10	4.31E-04	NS	-1.7	-1.5	-1.2	-1.1
Ephx1	epoxide hydrolase 1, microsomal (xenobiotic)	Proteolysis	1.60E-06	2.62E-02	NS	-1.8	-1.6	-1.2	-1.1
Ccdc85b	coiled-coil domain containing 85B	unknown	1.66E-05	4.54E-02	NS	-1.6	-1.4	-1.2	-1.1
A2m	alpha-2-macroglobulin	Protein transport	2.45E-10	3.68E-03	NS	-1.8	-1.7	-1.2	-1.1
Nono	non-POU domain containing, octamer-binding	mRNA processing	1.01E-06	3.80E-03	NS	-1.4	-1.3	-1.2	-1.1
Ifltd1	intermediate filament tail domain containing 1	unknown	6.75E-06	1.81E-04	NS	-1.2	-1.4	-1.2	-1.3
Myst4	MYST histone acetyltransferase (monocytic leukemia) 4	Transcription regulation	9.48E-06	1.18E-02	NS	-1.3	-1.3	-1.1	-1.2

**Genes down regulated in Meishan and up-regulated by ACTH**									

Gadd45b	growth arrest and DNA-damage-inducible, beta	Regulation of protein kinase activity	6.38E-04	1.41E-11	NS	-1.3	-1.3	2.4	2.4
C14orf4	chromosome 14 open reading frame 4	unknown	8.66E-03	7.41E-05	NS	-1.0	-1.2	1.4	1.2

Genes differentially expressed between Meishan (MS) and Large White (LW) pigs and/or in response to acute stimulation by ACTH were identified using a pig cDNA microarray made up of 8959 cDNA clones. The most significant affected genes (p < 0.0001) were listed in the table, categorized on the basis of up or down-regulated by genotype and/or ACTH and sorting by decreasing fold changes. (unknown transcripts were excluded) Complete list is given in the additional file 1.

### Quantitative analysis

Eleven genes highly significantly affected by genotype and/or treatment factors were selected for further examination by quantitative real-time PCR (Table 2). Changes in transcripts levels were confirmed for nine genes. Correlation coefficients between expression levels as measured with membrane hybridization and real-time PCR were significant (r^2 ^between 0.51 and 0.90, p < 0.05) for 9 out of 11 genes tested (see additional file 2). The magnitudes of the changes were roughly similar between the array and the real-time PCR except for the largest fold-changes (higher than 2 fold) that seemed dampened on the microarrays. An exception was *Rnf2 *gene, which showed no difference between genotypes of pigs or in response to ACTH when assayed by quantitative RT-PCR, but which exhibited a significant genotype difference when tested against the pig cDNA array. This disparity may have resulted from a false-discovery error or from cross-hybridization of transcripts to region of similarity in the arrayed *Rnf2 *cDNA. The second exception was *Fxc1*, which showed no significant difference when assayed by quantitative RT-PCR, but which exhibited a significant genotype difference when tested against the pig cDNA array. However, the fold changes between breeds found on the array were of low magnitude (< 1.5-fold).

**Table 2:** Measurement of genotype and/or ACTH effects on transcript accumulation by relative quantitative real-time PCR and comparison with microarray data

Gene symbol	Microarray							Real time PCR						
	
	p-value (ANOVA)			Fold Change				p-value (ANOVA)			Fold Change			
				
				genotype: MS/LW		treatment: ACTH/control					genotype: MS/LW		treatment: ACTH/control	
	
	genotype	treatment	interaction	control	ACTH	LW	MS	genotype	treatment	interaction	control	ACTH	LW	MS
Rnf2	2.81E-13	7.89E-01	1.96E-02	3.5	2.5	1.2	-1.2	0.82	0.64	0.81	1.1	-1.0	1.1	1.0
Anpep	5.65E-12	8.27E-03	NS	2.1	1.9	-1.1	-1.2	< 0.0001	0.040	0.056	3.9	3.0	-1.0	-1.4
Gadd45b	6.38E-04	1.41E-11	NS	-1.3	-1.3	2.4	2.4	0.028	< 0.0001	0.33	-1.5	-1.5	2.6	2.6
Eif1b	9.08E-06	7.89E-10	NS	1.5	1.9	2.5	3.3	0.009	< 0.0001	0.040	1.4	1.9	2.9	4.1
Crem	4.34E-02	2.70E-10	NS	1.7	1.1	4.4	3.0	0.18	< 0.0001	0.43	1.6	1.3	6.4	5.4
A2m	2.45E-10	3.68E-03	NS	-1.8	-1.7	-1.2	-1.1	< 0.0001	0.11	0.54	-2.1	-2.1	-1.3	-1.2
Ldlr*	5.19E-03	4.15E-07	NS	1.6	1.3	2.3	1.8	ND	ND	ND	ND	ND	ND	ND
Ckb	1.03E-06	3.00E-02	NS	2.1	1.9	1.3	1.2	0.004	0.43	0.88	2.3	2.1	1.3	1.2
Dab2	2.92E-02	5.63E-05	NS	-1.1	-1.3	-1.4	-1.7	0.34	0.052	0.63	-1.3	-1.2	-1.8	-1.6
Ldlr*	1.50E-02	1.14E-03	1.06E-02	1.5	1.0	1.6	1.1	0.069	< 0.0001	0.80	1.4	1.2	2.2	2.0
Fxc1	3.06E-04	2.35E-02	NS	1.2	1.3	1.1	1.1	0.28	0.16	0.48	-1.1	-1.4	1.6	1.2
Star	4.85E-04	NS	NS	1.6	1.2	1.4	1.1	0.053	0.26	0.406	1.4	1.1	1.3	1.0
Mc2r	ND	ND	ND	ND	ND	ND	ND	0.95	0.24	0.77	-1.1	1.0	1.2	1.4
Scarb1	NS	NS	NS	NS	NS	NS	NS	0.89	0.25	0.75	1.1	-1.0	1.2	1.1
Bzrp	ND	ND	ND	ND	ND	ND	ND	0.017	0.64	0.089	-2.3	-1.2	-1.4	1.4
Sqle	NS	NS	NS	NS	NS	NS	NS	0.16	0.39	0.32	-1.1	-1.5	1.3	-1.0
Cyp11a1	NS	NS	NS	NS	NS	NS	NS	0.85	0.36	0.37	1.2	-1.1	1.4	1.0
Hsd3b1	NS	NS	NS	NS	NS	NS	NS	0.51	0.17	0.94	-1.1	-1.1	-1.3	-1.3
Cyp21	NS	NS	NS	NS	NS	NS	NS	0.022	0.90	0.33	-1.2	-1.5	1.1	-1.1
Cyp11b	NS	NS	NS	NS	NS	NS	NS	0.13	0.27	0.94	1.3	1.3	1.2	1.2
Per2	ND	ND	ND	ND	ND	ND	ND	0.46	0.040	0.066	-1.2	1.8	-2.3	-1.0
Cry1	NS	NS	NS	NS	NS	NS	NS	0.96	0.48	0.62	1.1	-1.1	1.2	1.0
Bmal1	ND	ND	ND	ND	ND	ND	ND	0.002	0.21	0.70	-2.5	-2.9	-1.4	-1.6

The changes in expression levels for 11 genes shown by microarray analysis to be significantly regulated by genotype and/or ACTH were quantified by real-time PCR. For comparison, relative expression values for each gene were determined in each animal (fold change, n = 6 pigs per experimental point). Changes in expression levels were also analyzed for 11 additional genes of great interest for our study whereas they were either shown by microarray analysis to be not significant (NS, p > 0.05) or not determined (ND) since they were not included on the microarray or due to the data normalization process. * LDLR gene was represented by 2 cDNA clones on the microarray and changes in its expression were verified by real-time PCR using only the cDNA sequence for which exons/introns structure could be deduced.

Eleven additional genes involved in ACTH signaling, metabolism and mobilization of cholesterol, steroidogenesis and clock genes that were either not found to be significantly affected by genotype or ACTH treatment (*Scarb1*, *Sqle*, *Cyp11a1*, *Hsd3b1*, *Cyp21*, *Cyp11b*, *Cry1*) or not present on the array (*Mc2r*, *Bzrp*, *Per2*, *Bmal1*) were selected for further examination by quantitative real-time PCR because they were interesting for our study (Table 2). The absence of significant changes in the microarray study was confirmed for six out of seven genes.

The *Mc2r *gene that encodes the ACTH receptor was not present on the array. Quantitative real-time PCR showed that the *Mc2r *gene was not significantly affected by genotype or by ACTH treatment. Inasmuch as the present data showed that two transcripts involved in cholesterol transport (i.e. *Ldlr *and *Star*) were significantly affected by genotype and ACTH treatment, changes in the expression of *Scarb1 *and *Bzrp *genes, also involved in cholesterol transport, were measured by quantitative RT-PCR. Quantitative RT-PCR analysis showed that genotype and ACTH treatment did not affect the expression of *Scarb1*. However, the expression of *Bzrp *gene was found to be greater in Large White pigs whereas *Bzrp *was not found expressed on the array probably due to the low expression level of this gene and to the data normalization process used in the microarray analysis. *Sqle *is a key gene involved in an early step of cholesterol biosynthesis *de novo*. Its expression was not significantly affected by genotype or ACTH on the array. This was confirmed by real-time PCR. *Cyp11a1*, *Hsd3b1*, *Cyp21 *and *Cyp11b *that encode enzymes involved in steroidogenesis were unaffected by genotype or ACTH on the microarrays. Quantitative RT-PCR confirmed that neither genotype nor ACTH affected the levels of those genes except for *Cyp21*. While no significant difference was found when tested against the pig cDNA array, expression of *Cyp21 *was found to be greater in LW pigs when assayed by quantitative RT-PCR, but the fold changes were of low magnitude. Finally, 3 clock genes *Per2*, *Cry1 *and *Bmal1 *reported to be involved in circadian corticosteroids biosynthesis or adrenal responsiveness to ACTH were further investigated by quantitative real-time PCR. No significant change in *Cry1 *expression on the microarray was confirmed by real-time PCR. The two 2 other clock genes, *Per2 *and *Bmal1*, not present on the microarray were found significantly affected by ACTH treatment and genotype, respectively. *Per2 *was down-regulated in response to ACTH treatment and expression of *Bmal1 *was greater in LW pigs.

## Discussion

We used a comprehensive gene expression profiling by microarray analysis to identify groups of genes differentially expressed by genotype and/or by acute ACTH treatment. This is the first gene array analysis investigating *in vivo *adrenal response to an acute ACTH stimulation and exploring genetic variability at the adrenal level by using different breeds of pigs (i.e. an interesting biological model because pigs produce cortisol as humans). In microarrays, which included almost 8960 transcripts, the present results indicate that genotype and/or ACTH treatment affected the levels of 211 genes in adrenals. Moreover, although previous gene array analyses of ACTH action have been conducted *in vitro *and/or focused on the effects of chronic stimulation [25,27], our experiments demonstrate *in vivo *that acute ACTH treatment affects a large number of transcripts.

For the vast majority of affected transcripts, the changes were less than 2-fold excepted for some, probably of great interest, for which changes were as much as 4.5-fold. While some studies reported that small changes may be due to the tendency of microarray analysis to underestimate fold changes in transcripts accumulation [31], comparison of fold changes between microarray and real-time PCR analysis showed the accuracy of nylon microarrays used, even if fold-changes higher than 2-fold seemed to be somewhat underestimated. Our results suggest that genotypic difference and ACTH action may produce relatively small changes in transcript accumulation but these small changes could well be of physiological significance [32].

The dose of ACTH used in our study has been shown to maximally activate cortisol production within one hour [14,30] and thus, may produce maximum effects on steroidogenesis. Cortisol concentrations measured in our study were consistent with previous results reporting that cortisol levels induced by ACTH were higher in Meishans than in Large Whites, as well as basal cortisol levels [7,14]. However, ACTH did not affect gene expression of steroidogenesis enzymes (i.e. *Cyp11a1*, *Cyp17*, *3βhsd*, *Cyp21*, *Cyp11b*). This result is consistent with the fact that acute stimulation by ACTH had little effect on adrenal P450s and steroidogenic enzymes while in contrast, long-term ACTH treatment provokes profound changes in the mRNA levels of many adrenal steroidogenic enzymes [22,29,33]. Interestingly, no differences in expression of steroidogenesis genes were found between Meishan and Large White pigs. These results suggest that the difference of corticosteroidogenesis between Meishan and Large White pigs is not triggered by changes in gene expression of adrenal P450 and 3βHSD enzymes under basal state or following acute ACTH stimulation. Nevertheless, we cannot exclude that steroidogenic activity might be higher in Meishan pigs than in Large White pigs. Indeed, expression of several genes (*Mdh2*, *Sdha *and *Suclg2*) involved in the tricarboxylic acid (TCA) pathway was greater in MS pigs. The main catalytic function of TCA cycle is to provide reducing equivalents to the respiratory complexes [34], for example, steroid hydroxylation [35]. Moreover, the TCA cycle also contributes to the synthesis of heme [34], necessary for the prosthetic groups of the steroidogenic cytochrome P450s [36]. In this respect, it is worth noting that *Alas1*, the rate-limiting enzyme in heme biosynthesis [37], shows a greater expression in MS than in LW pigs (see additional file 1). These mechanisms may together result in a higher steroidogenic activity by supplying more reducing equivalents and heme to steroidogenic enzymes. This hypothesis is supported by previous observations indicating that heme availability limited adrenal corticosteroid biosynthesis [38] and by recent data showing that acute stimulation of steroid production by ACTH was significantly increased when heme oxygenase activity was inhibited [39].

The effects of ACTH are mediated through the ACTH receptor (MC2R) belonging to the melanocortin receptor family (MCRs). The binding of ACTH to its cognate G protein-coupled receptor promotes the activation of protein kinase A and MAPK-dependent signaling cascades that collectively initiate adrenal-specific steroidogenic transcriptional programs [21,22,40]. Findings from numerous *in vitro *studies support the notion that ACTH is a positive regulator of ACTH-R mRNA expression [33,41,42]. More particularly, Winnay and Hammer [43] demonstrated *in vitro *that ACTH stimulation acutely activates the *Mc2r *gene promoter (i.e. within 80 min). In our study, we showed that ACTH did not affect *Mc2r *gene expression within 1 hour and that this gene was not differentially expressed between MS and LW pigs. Similarly, no difference in gene expression encoding key regulator proteins of the ACTH signaling pathway (i.e. G protein, Adenylate Cyclase, Protein Kinase A and MAPK ERK1, ERK2) was found in our study. On the other hand, the levels of *Crem *(cAMP response element modulator), a cAMP-dependent transcription factor that functions to activate genes involved in steroidogenesis [44], were increased in presence of ACTH and higher in MS than LW pigs. Moreover, trophic hormone stimulation of steroidogenic cells has been shown to result in the activation of G proteins that stimulate adenylate cyclase activity and produce increased intracellular levels of cAMP [21]. Thus, high levels of *Crem *in MS compared to LW and in response to ACTH may result to a larger increase of signal transduction induced by ACTH.

A constant supply of cholesterol is required within adrenal cells for steroidogenesis. The rate-limiting step for steroidogenesis is the movement of unesterified cholesterol into mitochondria where it can then be metabolized by CYP11A1 and other enzymes in the steroidogenic pathway (for review see ref. [45]). The movement of cholesterol into the mitochondria is mediated by steroidogenic acute regulatory protein (StAR) [23] and other partners such as peripheral-type benzodiazepine receptor or translocator protein [46]. Interestingly, greater expression of the *Star *gene in Meishan than in Large White pigs found in our study suggests that enhanced cholesterol transport into mitochondria may contribute to the higher corticosteroid biosynthesis found in MS pigs compared to LW pigs. All studies on StAR function agree that this enzyme mediates acute stimulation of steroidogenesis in response to ACTH administration and requires *de novo *protein synthesis (for review see ref. [21]). However, we did not find changes in transcript levels of *Star *1 h following ACTH treatment while changes in *Star *gene expression induced by ACTH have been previously observed as early as 30 min and levels were maximally elevated between 1 and 3 h in the rat [29]. Nevertheless, phosphorylation of more preexisting StAR protein in MS pigs, a mechanism involved in the acute response to ACTH stimulation [47], could contribute to the higher cortisol levels induced in response to ACTH in MS than in LW pigs.

The unesterified cholesterol needed for steroidogenesis can be derived from several different sources (for review see ref. [45]). In our study we did not find differences in gene expression of enzymes involved in endogenous cellular cholesterol synthesis, such as *Sqle *or *Hmgcr*, between genotypes or in response to ACTH while those genes have been reported to be regulated by ACTH [25,27]. On the other hand, cellular cholesterol delivery for steroidogenesis includes uptake of lipoprotein-derived cholesterol via low density lipoprotein (LDL) receptor mediated endocytic pathways and SRB1 (Scavenger Receptor class B, type1) mediated "selective" pathways (for review see ref. [45]). Interestingly we found higher *Ldlr *expression in MS than in LW pigs and in response to ACTH but no changes were observed in *Scarb1 *expression.

Changes in *Ldlr *expression in response to ACTH found in our study are consistent with *Ldlr *up-regulation reported *in vitro *following 24 h ACTH treatment in Y1 mouse adrenal cells [25]. Conversely, while *Scarb1 *have also been reported to be up-regulated by 24 h ACTH treatment in Y1 mouse adrenal cells [25], and to a larger extent than *Ldlr*, we did not find change in *Scarb1 *expression. Our results indicate that receptor-mediated endocytic uptake of LDL-cholesterol may be a more important source of cholesterol for adrenal steroidogenesis in pigs as is the case in humans [48], while it appears to play a negligible role in mouse [45]. Moreover, this is the first evidence indicating *in vivo *that the acute response to ACTH may involve cellular cholesterol supply for steroidogenesis via *Ldlr *regulation.

A large number of genes found to be differentially expressed in our study encode transcription factors. Most of them have not been yet described as requisite in transcription networks involved in adrenal steroidogenesis. Nevertheless, they are potential interesting candidates, particularly those that were affected by both genotype and ACTH treatment. We were particularly interested in peripheral clock genes (such as *Bmal1*, *Per2*, *Cry1*), because recent studies reported that in the adrenal they regulate a large number of genes involved in general cellular processes (e.g. protein synthesis) as well as in pathways related to major organ-specific function (e.g. corticosteroid biosynthesis) and probably adjust adrenal sensitivity to ACTH [49-51]. Interestingly, while *Per2 *and *Cry1 *were not differentially expressed in our study, *Bmal1 *gene showed less expression in MS pigs. Thus, we can not exclude that differences in the expression level of some clock genes may be involved in differences in basal cortisol levels and adrenal reactivity to ACTH between MS and LW pigs. Further studies are needed to investigate other clock genes and to clarify how clock-controlled transcriptional rhythms in adrenals could contribute to the differences observed between both lines of pigs.

Phosphorylation and dephosphorylation mechanisms might be also involved in the difference of adrenal function between LW and MS pigs because a large number of differentially expressed genes encode diverse protein kinases and protein phosphatases. Among them *Snf1lk *(SIK1 protein) constitute a valuable candidate since it was reported to be an important regulator in the early phase of ACTH-signaling in the adrenal cortex [52].

## Conclusion

In conclusion, we report differential gene expression in adrenal in two lines of pigs in basal conditions and following acute ACTH treatment. Some of the genes have been already reported to be implicated in adrenal physiology, but the majority has not been documented as directly involved in steroidogenesis regulation or as acutely ACTH-responsive. Although their contributions to adrenal function merit further investigation, we may speculate on the involvement of a few of them. The higher cortisol levels in basal state and in response to ACTH in MS than in LW pigs was probably not triggered by changes in gene expression of known key regulator proteins of the ACTH signaling pathway and steroidogenic enzymes. However, a higher TCA cycle activity in MS pigs than in LW pigs may explain the higher steroidogenic activity by supplying more reducing equivalents and heme to steroidogenic enzymes. Alternately, differences in the adrenal function between MS and LW pigs involve likely mechanisms requisite for cholesterol supply to steroidogenesis. The genes described in this report are thus excellent potential candidates to mediate the genetic differences in adrenal steroidogenesis, particularly those affected by genotype and ACTH. Because dysregulation of glucocorticoid production results in diverse diseases, elucidation of the function of these genes in adrenals will lead to better understanding the molecular basis of such pathological conditions.

## Methods

### Animals and housing

Seven-week-old male Large White (LW) and Meishan (MS) piglets (n = 24) were used in this study. The animals were reared and experiments were conducted at the INRA experimental research farm of Le Magneraud (Surgères, France). Piglets were weaned at four weeks of age and then allocated into groups of 24 animals in 3.65 × 1.70 m pens. Animals were housed in collective pens on a 10 h light, 14 h dark cycle (natural photoperiod) with food and water *ad libitum*. Experimental groups of six pigs of both genotypes were randomly constituted and placed in collective pens one week before the experiments. For each genotype, pigs used in this study were taken from five litters resulting from matings with two boars. The experiments described here fully comply with legislation on research involving animal subjects according to the European Communities Council Directive of November 24, 1986 (86/609/EEC). Investigators were certificated by the French governmental authority for carrying out these experiments.

### Treatment and sampling

Piglets were either non-treated or injected in the neck muscle with mammalian 1–24 ACTH (Immediate Synachten, Novartis France) at the dose of 250 μg per animal. The dose of ACTH was chosen to ensure a maximum cortisol release [30]. Non-treated animals have been chosen as control instead of animals injected with vehicle in order to have the most accurate basal conditions. In accordance with approved slaughter methods, piglets were stunned and immediately exsanguinated after capture from their home pen (non-treated animals) or one hour following ACTH injection. Experiments were performed between 08.00 h and 10.00 h. Blood samples were collected directly from each piglet in heparined tubes at sacrifice. The blood was kept on ice until centrifugation (4000 g for 10 min) and plasma frozen at -80°C until measurement of cortisol. The adrenal glands were also collected, frozen immediately on dry ice and then stored at -80°C until RNA isolation.

### Cortisol measurement

Plasma total cortisol was measured using a specific radio immunoassay (as previously described in Désautés *et al *[7]). The cortisol data were transformed to base 10 logarithmic scores and analyzed by ANOVA to assess the effects of genotype, treatment and their interaction. Results are given as the mean ± standard error.

### Total RNA isolation and purification

For each biological sample, entirely left adrenal gland was homogenized in TRIzol reagent (Invitrogen Life Technologies) and a part of the homogenized sample was then used for total RNA isolation, followed by column purification (RNeasy MinElute kit, Qiagen). This procedure ensures to get equal proportions of cortex and medulla between samples. DNA was digested using an RNase-free DNase set (Qiagen) during RNA purification. Total RNA was quantified by spectrophotometer (NanoDrop^®^) and its integrity was assessed on an Agilent 2100 Bioanalyser (RNA 6000 Nano LabChip, Agilent Technologies).

### Microarray analysis

Gene expression was analyzed by hybridization of non commercial nylon cDNA microarrays (accession in Gene Expression Omnibus data sets: GPL3729) developed by the Biological Resources Center GADIE (Genomic for animals of economical importance, INRA France) and consisted of PCR products from 8959 cDNA clones [53]. cDNA from luciferase was present on the array as positive control (193 spots) and water was also included as negative control (64 spots). cDNA clones came from pig normalized multi-tissues libraries including adrenal glands collected from control and stress pigs. Microarrays were first hybridized with a ^33^P-labeled oligonucleotide sequence common to all PCR products to control the quality of spotting and quantity of target DNA accessible in each spot. Microarrays were then hybridized with ^33^P-labeled complex probes synthesized and labelled from 5 μg of total RNA with Supersript II RNAse H- reverse transcriptase (Invitrogen). mRNA from luciferase were added to the pigs samples for calibration. Hybridizations were carried out during 24 hours at 68°C. After washing, arrays were exposed for six to 12 hours to radioisotopic-sensitive imaging plates. Detection scanning was done with a FUJI BAS 5000 phosphoimager (Fujifilm) at 25-μm resolution and quantification of hybridization signals with the AGScan software [54]. One microarray hybridization per animal was done giving six biological replicates per experimental point.

### Microarray data normalization and statistical analysis

Before statistical analysis, data were log_10 _transformed and centred by median for each array and each gene. A filter procedure eliminated non informative transcripts on the basis of being well measured (expression level > mean + 2 standard deviations of background signal) in 100% of the samples. Statistical analyses were done using the R software (version 2.2.1, [55]). A linear model was used to test the effect of genotype and treatment as well as their interaction, and variation in quantity of target DNA accessible in each spot was included as covariate. False discovery rate (FDR) was determined using the Benjamini-Hochberg procedure [56].

The microarray data from this research has been deposited in the NCBI Gene Expression Omnibus data repository under accession number GSE8377[57].

### Functional annotation

Transcripts significantly affected by genotype and/or ACTH treatment were annotated for their function according to Gene Ontology database [58] and Expression Analysis Systematic Explorer (EASE) software from DAVID bioinformatics database [59].

### Analysis of RNA changes by relative quantitative real-time PCR

To verify changes in gene expression, real-time PCR was carried out on 22 selected genes. RNA (4 μg) was reverse transcribed in a total volume of 20 μl using 200U of Superscript II (Invitrogen) reverse transcriptase, 100 pmol oligo-dT_22_V, 0.5 mM deoxy-NTP, and 40U RNasin (Promega). The resultant cDNA was diluted 1:100 with nuclease-free water. Five microliters of diluted cDNA was used in subsequent PCR reactions. All primers were designed based on nucleotide sequences in Genbank using the Primer Express software (PE Applied Biosystems) (table 3). PCR reaction efficiency was calculated for each primer pair with five dilution points of the calibrator sample to validate primers. Introns-exons organisation of the porcine genes was deduced by comparison with human genes using ICCARE software and primers from one pair were chosen in distinct exons of the corresponding gene. Each real-time PCR reaction consisted of 1× Power SYBR Green PCR Master Mix (PE Applied Biosystems), 0.5 μM forward and reverse primers and cDNA to a total volume of 20 μl. Reactions were carried out on an ABI PRISM 7500 Sequence detection system (PE Applied Biosystems) for 40 cycles (95°C for 15 s, 60°C for 1 min). The fold change in expression of each gene was calculated using the ΔΔCt method with the levels of transketolase RNA as an internal control; as determined by quantitative RT-PCR, the levels of transketolase did not change depending on genotype or treatment in our study (data not shown) and transketolase gene has previously been used to normalize data from quantitative RT-PCR in adrenal cells [25]. Quantitative real-time PCR analysis was done in each out of the six animals constituting an experimental point and measurements were done in duplicate. ANOVA was conducted on relative expression to assess the effects of genotype, treatment and their interaction.

**Table 3:** Gene-specific primers used for quantitative RT-PCR

Gene symbol	Forward Primer	Reverse Primer	accession no.
Dab2	CCCGTGATGTGACAGACAACC	ACTAATGGCTCTGCCTGTTGC	BX918681
A2m	ACGTGAGCCGAACAGAGGTC	GCGATGGCAAACTCAGCTG	BX674240
Anpep	CTCATTCGGAAGCAAGACGC	CCACCGCCATAGTCCTGAAA	BX665286
Fxc1	TCCTTCCAGGAGGCCTGTC	GCTGTACCAGGGCAGGCAT	BX674767
Rnf2	CACTGTGTTAAATGGCTCTTTTTCTT	TGTGCTCCTTTGTGGGTGC	BX673517
Gadd45b	GCTGATGAATGTGGACCCTGA	CCTGACACCCGCACGATATC	BX671980
Eif1b	GTTTCTCTTGGAGGTTGGCATT	TCACGAGGCAGCCAAACTG	BX926052
Crem	AACACGCAAACGAGAGCTGAG	GCACAGCCACACGGTTTTC	BX670994
Tkt	GGACAGGAAGCTCATTCTCGA	AGCAGCCACTGCCTCACCTA	BX925610
Star	GAAGAGCTTGTGGAGCGCAT	AGCCAACTCATGGGTGATGAC	U53020
Scarb1	TGTGGTTTGCAGAGAGCGG	ATGAACAGCAGGACGCAGC	NM_213967
Cyp21	TGCTTCACCACCCTGAGATTC	GCCCAGCTCGCGATCTAAC	BX916139
Cyp11a1	AGACACTGAGACTCCACCCCA	GACGGCCACTTGTACCAATGT	BX674077
Ldlr	GCCTCACAGGCTCGGACATA	ACACCAGTTCACCCCTCTCG	BX673438
Sqle	TGGTCCAGTTGCGCTGATT	GGGCTCCGATTTAAAGCAAAA	BX920102
Bzrp	GGCACACTCTACTCGGCCAT	ACAGCCTCCTCCGAGAAGCT	BX925849
Ckb	TTCACCCGCTTCTGCAATG	AGGTCAGGATGTAGCCCAGGT	BX920566
Hsd3b1	TTCCGCCCTCTCTGAGGTACT	GGTCACGAAGTGGCGATTG	BX919321
Bmal1	TCCTAGCCAACGTCCTGGAA	TCTTTGGGCCACCTTCTCC	EF216896
Cry1	TGAACCACGCTGAGGCAAG	GGATTAGATGGCACTGACGCA	BQ600826
Per2	GACGTGCCGGAATGTGTTTAC	GCTCCCGGTTTCTGTGACTC	CF789448
Mc2r	ACCATGTCCCCGCAGTGAT	GTGATGGCCCCTTTCATGTT	AF450083
Cyp11b	CCCGTGGGTATCTTCTTGGA	GGTTTCGACCCAGGGAGTAGA	D38590

Gene-specific primers were designed based on sequences provided under the accession numbers listed in the National Center for Biotechnology Information (NCBI) database.

## Abbreviations

ACTH, adrenocorticotropic hormone; Alas1, aminolevulinate delta synthase 1; Bmal1 (or Arntl), Brain and muscle ARNT-like 1 (or aryl hydrocarbon receptor nuclear translocator-like protein 1); Bzrp (or TSPO), peripheral-type benzodiazepine receptor (or translocator protein); CBG, corticosteroid-binding globulin; Crem, cAMP responsive element modulator; CRH, corticotropin-releasing hormone; Cry1, cryptochrome 1; Cyp11a1, cytochrome P450 family 11 subfamily B polypeptide 1; Cyp11b, cytochrome P450 family 11 subfamily B; Cyp17, cytochrome P450 family 17; Cyp21, cytochrome P450 family 21; ERK, extra-cellular signal regulated kinase; Fxc1, fracture callus 1 homolog (rat); Hmgcr, hydroxymethylglutaryl coenzymeA reductase; HPA, hypothalamic-pituitary-adrenal; 3βhsd (or Hsd3b1), 3β hydroxysteroid dehydrogenase; Ldlr, low density lipoprotein receptor; LW, Large White; MAPK, mitogen activating protein kinase; Mc2r, melanocortin 2 receptor; Mdh2, malate dehydrogenase 2 NAD (mitochondrial); MS, Meishan; P450scc, cytochrome P450 cholesterol side chain cleavage enzyme; Per2, period homolog 2; Rnf2, ring finger protein 2; Scarb1, scavenger receptor beta 1; Sdha, succinate dehydrogenase complex subunit A flavoprotein (Fp); Snf1lk, SNF1-like kinase; Sqle, squalene epoxidase; Star, steroidogenic acute regulator; Suclg2, succinate-CoA ligase GDP-forming beta subunit; TCA, tricarboxylic acid.

## Authors' contributions

DH was involved in experimental design and in planning the study, carried out experiments on animals, performed radioimmunoassay, extracted RNA, carried out microarray molecular work and real-time PCR work, performed statistical analysis, interpreted data and drafted the paper. LL was involved in the microarray molecular work, contributed to the statistical analyses of the microarray data and to the writing of the manuscript. MSC designed and performed statistical analyses of microarray data and contributed to the writing of the manuscript. PM conceived of the study and coordinated the experimental design, carried out experiments on animals, contributed to the RNA extraction and to the microarray molecular work, to the analysis and interpretation of data, and to writing the paper. All authors have read and approved the final manuscript.

## Supplementary Material

Additional file 1 Complete list of genes differentially expressed depending on genotype and/or ACTH treatment. The data include one table. Click here for file

Additional file 2 Means and standard errors (SE) of transcripts levels (log) and Pearson correlation coefficients between real-time PCRs and microarray data. The data include one table. Click here for file
